# Development of novel monoclonal antibodies with specific binding affinity for denatured human CD26 in formalin-fixed paraffin-embedded and decalcified specimens

**DOI:** 10.1371/journal.pone.0218330

**Published:** 2019-06-13

**Authors:** Ryo Hatano, Taketo Yamada, Hiroko Madokoro, Haruna Otsuka, Eriko Komiya, Takumi Itoh, Yuka Narita, Satoshi Iwata, Hiroto Yamazaki, Shuji Matsuoka, Nam H. Dang, Kei Ohnuma, Chikao Morimoto

**Affiliations:** 1 Department of Therapy Development and Innovation for Immune Disorders and Cancers, Graduate School of Medicine, Juntendo University, Hongo, Bunkyo-ku, Tokyo, Japan; 2 Department of Pathology, Saitama Medical University, Moroyama-machi, Iruma-gun, Saitama, Japan; 3 Department of Pathology, Keio University School of Medicine, Shinjuku-ku, Tokyo, Japan; 4 Department of Immunological Diagnosis, Juntendo University Graduate School of Medicine, Hongo, Bunkyo-ku, Tokyo, Japan; 5 Division of Hematology/Oncology, University of Florida, Gainesville, FL, United States of America; Wayne State University, UNITED STATES

## Abstract

A 110-kDa type II transmembrane glycoprotein with dipeptidyl peptidase IV (DPPIV) activity in its extracellular region, CD26 has a multitude of biological functions and plays an important role in the regulation of inflammatory responses and tumor biology. Our work has focused on CD26 as a novel therapeutic target for various tumors and immune disorders, and we have recently developed a humanized anti-CD26 monoclonal antibody (mAb), YS110, which has promising safety profile and clinical activity in patients with malignant pleural mesothelioma. The development of an anti-human CD26 mAb that can clearly and reliably detect the denatured CD26 molecule in formalin-fixed paraffin-embedded (FFPE) tissues in the clinical setting is therefore of the utmost importance. To develop novel anti-CD26 mAbs capable of binding to denatured CD26, we immunized mice with urea-treated CD26 protein. Hybridoma supernatants were screened for specific reactivity with human CD26 by immunostaining through the use of a set of FFPE human CD26-positive or negative tumor cell lines. This screening method enables us to develop novel anti-human CD26 mAbs suitable for immunohistochemical staining of CD26 in FFPE non-tumor and tumor tissue sections with reliable clarity and intensity. Specifically, these mAbs display strong binding affinity to denatured human CD26 rather than undenatured human CD26, and are capable of detecting denatured human CD26 in decalcified specimens. These novel anti-CD26 mAbs are potentially useful for the analysis of CD26 expression in cancer patients with bony metastasis, and may help decide the appropriateness of YS110 therapy for future cancer patients.

## Introduction

CD26 is a homodimeric type II transmembrane glycoprotein with a molecular mass of 220–240 kDa [1, 2]. Human CD26 is composed of 766 amino acids (AAs), including a short cytoplasmic domain of 6 amino acid residues at the N-terminal end (AA 1–6), a transmembrane region of 22 amino acids (AA 7–28), and an extracellular domain, the predominant part of CD26 (AA 29–766) [3, 4]. This C-terminal extracellular domain exhibits dipeptidyl peptidase IV (DPPIV) activity. DPPIV belongs to the serine protease family, able to cleave dipeptides from polypeptides with N-terminal penultimate proline or alanine, and regulates biological activities of a number of mediators such as cytokines, chemokines, neuropeptides and incretin hormones [5]. Although CD26 is expressed on various cell types, including epithelial cells (kidney proximal tubules, bile duct, prostate and intestine), endothelial cells as well as T lymphocytes [3, 4, 6], its function is dependent on cell types and the microenvironment, which influences the multiple biological roles of CD26.

CD26 is expressed on both human CD4^+^ T cells and CD8^+^ T cells in a triphasic fashion, and is associated with T cell signal transduction processes as a costimulatory molecule [7, 8]. In addition to its function as an immunoregulatory molecule, CD26 is expressed on a number of human neoplasms, including malignant pleural mesothelioma (MPM), renal carcinoma (RCC), colorectal cancer (CRC), hepatocellular carcinoma, lung cancer, prostate cancer, gastrointestinal stromal tumor (GIST), thyroid carcinoma, and different subtypes of T-cell malignancies [9]. Although CD26/DPPIV function in cancer biology is not yet fully characterized, CD26 serves as a prognostic marker in multiple tumors such as CRC, GIST, thyroid carcinoma, urothelial carcinoma and prostate cancer [10–14]. Moreover, in several human malignancies including MPM, CRC and chronic myeloid leukemia, CD26/DPPIV expression is reported to be a marker of cancer stem cells [15–18]. Besides our work over the past three decades characterizing its role in the immune system, our group has also had a long-standing interest in the role of CD26 in cancer biology. We have previously demonstrated that anti-CD26 monoclonal antibody (mAb) treatment resulted in both *in vitro* and *in vivo* inhibition of tumor cell growth, migration and invasion, and enhanced survival of mouse xenograft models inoculated with T-cell lymphoma, RCC or MPM via multiple mechanisms of action [19–23]. These findings have led to our focus on CD26 as a novel therapeutic target for various tumors, and the development of YS110, a humanized mAb with high affinity to the CD26 antigen. Results from the first-in-human (FIH) phase I clinical trial of this mAb for CD26-expressing solid tumors, particularly refractory MPM, were recently published [24]. Our FIH study demonstrated that YS110 therapy exhibited a favorable safety profile and resulted in encouraging disease stabilization in a number of patients with advanced/refractory MPM and RCC. A subsequent phase II clinical trial of YS110 for MPM is currently in progress in Japan [25].

Along with the development of novel targeted therapies that can be administered at an optimal dose and schedule to maximize efficacy with tolerable toxicities is the acute need for the concurrent development of accurate companion diagnostic agents to select the appropriate patient population for treatment. It is therefore imperative to develop a detection method for CD26 expression in formalin-fixed paraffin-embedded (FFPE) clinical tumor samples that allows for the selection of potentially eligible patients in the clinical setting for humanized anti-CD26 mAb therapy. Despite our extensive testing of the many anti-CD26 mAbs previously developed in our laboratory [26] and the 23 commercially available anti-CD26 mAbs, none of them can clearly detect the denatured CD26 molecule in FFPE tissues. On the other hand, we have tested 5 commercially available anti-CD26 polyclonal antibodies (pAbs), and among them, a pAb purchased from R&D Systems showed that these reagents exhibited the most reliable staining pattern and intensity [24, 27, 28]. However, the potential lot-to-lot variability in staining pattern and intensity and the general lack of product uniformity represent shortcomings for the use of pAbs in the clinical setting. These inconsistencies and the difficulty in maintaining a stable supply hence make pAbs not the ideal reagents for diagnostic testing of patient tumor samples. For these reasons, we recently attempted to develop novel anti-human CD26 mAbs by immunizing mice with urea-treated CD26 protein, and succeeded in developing a mAb, clone 19–32, capable of detecting denatured CD26 in FFPE tissue sections with reliable intensity [29]. However, in the process of developing the companion diagnostic kit utilizing our 19–32 mAb for clinical usage, the critical issue involving non-specific immunostaining of control slides has unexpectedly arisen. 19–32 mAb stained not only CD26-positive tumor cell line specimens, but also those from CD26-negative tumor cell lines as well, strongly suggesting that it is inappropriate for the detection of denatured CD26 expression in FFPE clinical tumor samples.

In the present study, to address this critical issue, we have improved the screening methods and succeeded in developing novel anti-human CD26 mAbs with strong binding affinity to denatured human CD26 in FFPE non-tumor and tumor tissue sections, and which do not stain CD26-negative specimens, suggesting that these novel mAbs are potentially useful for the analysis of CD26 expression in cancer patients, and may help decide the appropriateness of YS110 therapy for future cancer patients.

## Materials and methods

### Animals

Female BALB/c mice were purchased from CLEA Japan (Tokyo, Japan) and female CB17/lcr-*Prkdc*^*scid*^/CrlCrlj mice (SCID mice) were purchased from Charles River Laboratories Japan, Inc. (Yokohama, Japan). Mice were housed in a specific pathogen-free facility in micro-isolator cages with ad libitum access to autoclaved water and sterile standard food, and were maintained at 24 ± 2°C under a 12-hour light-dark cycle (lights on 8 am to 8 pm). All mice were used at 6–8 weeks of age. To minimize suffering and distress, subcutaneous injection in the flank of mice was performed under isoflurane anesthesia through an isoflurane vaporizer set to deliver 2–3% isoflurane.

### Cell lines and cultures

A human MPM cell line MSTO-211H (MSTO parent), a human lung cancer cell line A549 and a human T-cell leukemia line Jurkat (Jurkat parent) were obtained from the American Type Culture Collection (Rockville, MD). MSTO parent cells were stably transfected with a full-length human CD26 (MSTO-CD26) using the Lipofectamine2000 reagent (Invitrogen, Carlsbad, CA), and selected with G418 (Sigma-Aldrich, St Louis, MO) [23]. The human MPM cell line JMN was a kind gift from Dr. Brenda Gerwin (Laboratory of Human Carcinogenesis, National Institutes of Health, Brethesda, MD). JMN cells were transduced with the short hairpin RNA (shRNA)-expressing lentivirus (MISSION; Sigma-Aldrich), and the stable cell lines (JMN CD26-shRNA, JMN ctrl-shRNA) were generated by selection with puromycin [16]. Jurkat parent cells were stably transfected with a full-length human CD26 by electroporation (Jurkat-CD26), and generated by selection with G418 [2]. All cell lines were grown in RPMI1640 medium supplemented with 10% FBS at 37°C in a humidified 5% CO_2_ incubator.

### Antibodies

To characterize the newly developed mouse anti-human CD26 mAbs, murine anti-human CD26 mAbs (clone 1F7, 5F8 or 19–32) which were previously developed in our laboratory were used for comparison [7, 29, 30]. To compare the staining pattern and intensity of human CD26 on FFPE tissue or cell block, we used a purified goat anti-human CD26 pAb (AF1180) purchased from R&D Systems (Minneapolis, MN) as a control.

### Preparation of immunogen

Soluble human CD26 (sCD26) was produced according to the method described previously [29, 31]. Purified sCD26 was denatured in 8 M urea buffer supplemented with 20 mM HEPES and 50 mM dithiothreitol (DTT) by gentle rotation for 8 hours at RT.

### Development of hybridomas and monoclonal anti-human CD26 antibodies

The methods for the development of hybridoma were detailed previously [29]. Hybridoma supernatants were first screened for murine IgG production by enzyme-linked immunosorbent assay (ELISA) utilizing Mouse IgG total Ready-SET-Go! ELISA set (eBioscience, San Diego, CA). The supernatants containing high levels of murine IgG (more than 2 μg/ml) were next screened for immunostaining of FFPE cell block and tissue sections. The hybridomas were cloned by limiting dilution. Monoclonal antibodies were purified from the supernatants using Protein A IgG Purification Kit (Pierce, Rockford, IL).

### Immunostaining of cell block or tissue specimens

The cultured cell lines were fixed in 10% neutral buffered formalin for 48 hours at RT, and subsequently embedded in paraffin. MSTO parent, MSTO-CD26 or JMN cells (1 x 10^6^, each) were implanted subcutaneously (s.c.) in the flank of SCID mice. Tumor volumes were measured along three orthogonal areas (a, b and c) by digital vernier calipers (Mitutoyo Corporation, Kanagawa, Japan), and calculated as tumor volume = abc/2 (mm^3^). Tumor volumes were monitored twice a week, and after 8 weeks of implantation (when the tumor volume reached around 500 mm^3^), mice were sacrificed by cervical dislocation and the tumors in the flank were excised and fixed in 10% neutral buffered formalin at RT, and subsequently embedded in paraffin. In this experiment, tumor size was not sufficiently large to impact survival, overall weight and locomotor activity of mice. FFPE tissue specimens of human MPM and normal liver, kidney and prostate were used for positive controls. For the analysis of normal human bone and bone marrow tissue, or bone with metastatic cancer, formalin-fixed samples were decalcified in 10% formic acid at RT or 10% EDTA in Tris buffer, and subsequently embedded in paraffin. FFPE cell block or tissue specimens were cut into 4–6 μm sections and deparaffinized. For histology, sections were stained with hematoxylin and eosin. The methods for immunohistochemistry were detailed previously [29]. To confirm the binding specificity of mAbs to human CD26, 100 μl of anti-human CD26 mAb solution (10 μg/ml in PBS) was gently rotated with 1 mg/ml of sCD26 at 4°C overnight, and after centrifugation, the supernatant was used instead of the primary anti-human CD26 antibody (Ab).

### Western blotting

The cultured cell lines were collected and lysed in RIPA buffer supplemented with 2% protease inhibitor cocktail (Sigma-Aldrich) for 1 hour at 4°C. Following addition of Laemmli 4x SDS sample buffer and DTT, whole cell lysates were boiled for 5 min at 95°C. Each sample was electrophoretically separated on 4–20% Mini-PROTEAN TGX precast gels (Bio-Rad, Hercules, CA) with Tris/glycine buffer, and transferred to PVDF membrane. After blocking with 5% skim milk in TBS for 45 min at RT, membranes were incubated with purified mouse anti-human CD26 mAb (19–32: 5 μg/ml, U16-3: 0.2 μg/ml, U38-8: 0.5 μg/ml) or goat anti-human CD26 pAb (1 μg/ml) in 5% skim milk-TBS at 4°C overnight. The membranes were washed with TBS containing 0.1% Tween-20 (TBS-T) and incubated with horseradish peroxidase (HRP)-conjugated sheep anti-mouse IgG Ab (GE Healthcare, Buckinghamshire, UK) or HRP-conjugated donkey anti-goat IgG Ab (Santa Cruz Biotechnology, Santa Cruz, CA) in 5% skim milk-TBS for 1 hour at RT. Proteins were detected with enhanced chemiluminescence using the Western Lightning Plus-ECL (Perkin Elmer, Waltham, MA). The images were taken using Luminescent Image Analyzer LAS-4000 (GE Healthcare) and data were analyzed with Image Reader LAS-4000 and Multi Gauge software (GE Healthcare). For reprobing, the same membranes were treated with stripping solution. After blocking with 5% skim milk in TBS, the membranes were reblotted with mouse anti-β-actin mAb (clone AC-15, Sigma-Aldrich) in 5% skim milk-TBS for 1 hour at RT, and subsequently incubated with HRP-conjugated sheep anti-mouse IgG Ab in 5% skim milk-TBS for 1 hour at RT. Protein detection was performed as described above.

### Flow cytometry

The methods for flow cytometry were detailed previously [29]. In brief, The cultured cell lines were incubated with 10 μg/ml of purified mouse anti-human CD26 mAb (19–32, U16-3 or U38-8) or goat anti-human CD26 pAb or isotype controls for 25 min at 4°C, and subsequently stained with PE-conjugated goat anti-mouse Ig pAb (BD Biosciences, San Jose, CA) or PE-conjugated donkey anti-goat IgG Ab (Santa Cruz Biotechnology) for 25 min at 4°C. PE-labeled mouse anti-human CD26 mAb (clone M-A261, BD Biosciences) was utilized as a positive control. PE-labeled or unlabeled mouse IgG_1_,κ isotype control (clone MOPC-21, BD Biosciences) and normal goat polyclonal IgG (AB-108-C, R&D Systems) were utilized as negative controls.

### ELISA

For the preparation of denatured sCD26 used for ELISA, purified sCD26 protein was boiled for 10 min at 95°C in PBS. The 96-well immunoplates (NUNC, Roskilde, Denmark) were coated with native (undenatured) sCD26 or denatured sCD26 in carbonate bicarbonate buffer (12.5, 25, 50, 100, 200 ng/well, respectively) or buffer alone as a negative control at 4°C overnight. Each well of the plate was blocked with 2% BlockAce (DS Pharma Biomedical, Osaka, Japan) in deionized distilled water for 1 hour at RT, and then incubated with 2 μg/ml of purified mouse anti-human CD26 mAb (5F8, 1F7, 19–32, U16-3 or U38-8) or goat anti-human CD26 pAb in 1% BlockAce solution for 1 hour at RT, and subsequently incubated with HRP-conjugated goat anti-mouse Ig pAb (BD Biosciences) or HRP-conjugated donkey anti-goat IgG Ab (Santa Cruz Biotechnology) in 1% BlockAce solution for 1 hour at RT. Colorimetric methods and data analysis were detailed previously [29].

### Ethics approval and consent to participate

Animal experiments were conducted following protocols approved by the Animal Care and Use Committee at Juntendo University (Authorization Numbers: 250170 and 290131). For the use of human materials, experimental procedures and study protocols were approved by the Saitama Medical University ethical review board (Authorization Number: 794), the Keio University School of Medicine ethical review board (Authorization Number: 2012–0100) and the Juntendo University School of Medicine ethical review board (Authorization Number: 2014029), and the purpose of the study was explained to all patients and their written informed consent was obtained. The use of human samples from autopsy cases was generously permitted by the written informed consent obtained from the next of kin. All studies on human subjects were carried out according to the principles set out in the Declaration of Helsinki.

## Results

### Development of novel anti-CD26 mAbs

**Fig 1:**
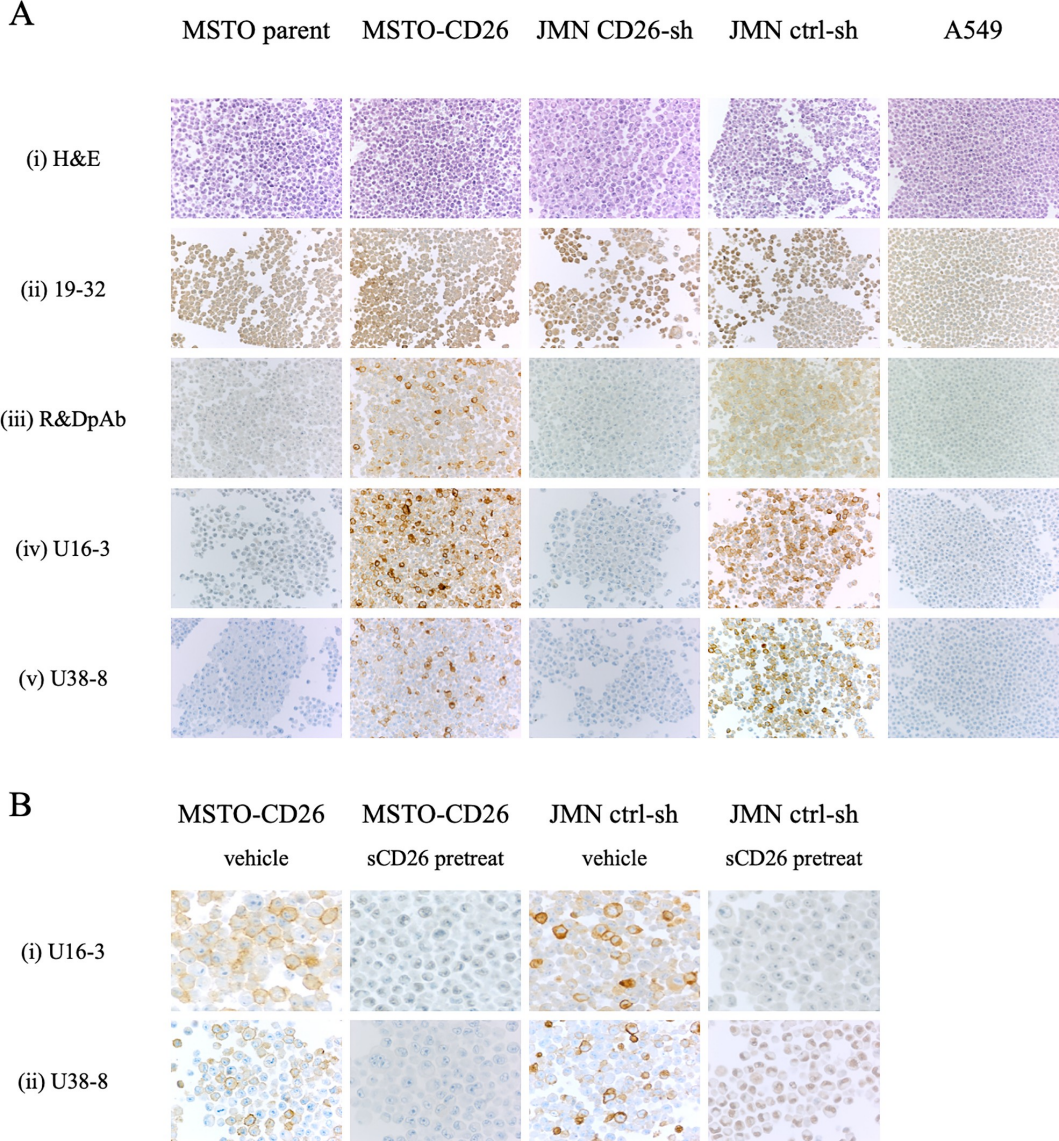
Representative results of immunostaining of FFPE cell block with novel anti-CD26 mAbs. **A.** The cell block sections of MSTO parent, MSTO-CD26, JMN CD26-shRNA, JMN ctrl-shRNA or A549 cells were stained with hematoxylin and eosin (H&E) (i), or purified mouse anti-human CD26 mAb (19–32 (ii), U16-3 (iv), U38-8 (v)), or purified goat anti-human CD26 pAb (R&D Systems (iii)). Original magnification, 10x. **B.** The cell block sections of MSTO-CD26 or JMN ctrl-shRNA cells were stained with purified novel mouse anti-human CD26 mAbs (U16-3 (i) or U38-8 (ii)) in the presence of excessive amounts of soluble human CD26 protein (sCD26) or vehicle. Original magnification, 40x. All specimens were counterstained with hematoxylin.

Following the development of a mouse anti-human CD26 mAb, clone 19–32, capable of detecting denatured CD26 in FFPE tissue sections [29], we attempted to establish the companion diagnostic kit utilizing this mAb. The control slides containing samples from a set of FFPE cell lines expressing CD26 or CD26-negative cell lines are included in the kit as positive or negative controls for immunostaining, respectively. For this purpose, we selected the human CD26-negative lung cancer cell line A549 and 3 pairs of human CD26-positive or negative tumor cell lines (MSTO, JMN and Jurkat cells) which have been previously characterized by our group [2, 16, 23]. To confirm mRNA and cell surface protein expression of human CD26 in these cell lines, we conducted real-time RT-PCR and flow cytometry analyses. MSTO parent, A549 and Jurkat parent were endogenous human CD26-deficit cell lines, and neither mRNA nor cell surface protein expression was detected in these cells, while both MSTO-CD26 and Jurkat-CD26 which were stably transfected with a full-length human CD26 expressed CD26 at both mRNA and protein levels (S1A and S1B Fig). On the other hand, stable shRNA knockdown of CD26 in JMN, an endogenous human CD26-positive cell line, markedly reduced the expression at both mRNA and protein levels as compared with JMN ctrl-shRNA cells (S1A and S1B Fig). We next prepared the FFPE cell block of these tumor cell lines (the results of H&E staining of each tumor cell line were shown in Fig 1A-i), and examined immunostaining with 19–32 mAb. However, although FFPE tissue specimens of normal liver, kidney, prostate, and malignant mesothelioma stained with 19–32 mAb exhibited reliable staining pattern and intensity [29], specimens from both CD26-positive MSTO-CD26 and JMN ctrl-shRNA cells as well as CD26-negative MSTO parent, JMN CD26-shRNA and A549 cells were all unexpectedly stained with 19–32 mAb (Fig 1A-ii). Meanwhile, cell block specimens stained with anti-CD26 pAb purchased from R&D Systems (control pAb) exhibited a clear staining pattern of CD26, (Fig 1A-iii), revealing 19–32 mAb to be inappropriate for the detection of denatured CD26 expression in FFPE clinical tumor samples.

Given these observations, we again attempted to develop novel anti-human CD26 mAbs that are useful for the analysis of tumor CD26 expression in the clinical setting. To achieve this objective, we improved the hybridoma screening methods that are summarized in Table 1.

**Table 1:** A method for development of novel anti-human CD26 monoclonal antibodies.

	Host	Immunogen	1st screening	2nd screening	3rd screening
The previous method (19–32 mAb)	BALB/c mice	8M urea-treated recombinant sCD26^a^ (protein)	**Flow cytometry** Jurkat-CD26 (CD26^+^) Jurkat parent (CD26-)	**ELISA** Native sCD26^a^ Urea-treated sCD26^a^	**Immunostaining** [Tissue] Liver Kidney Prostate Mesothelioma
The present method (U16-3 mAb, U38-8 mAb)	BALB/c mice	8M urea-treated recombinant sCD26^a^ (protein)	**ELISA** Mouse total IgG	**Immunostaining** [Cell line] MSTO parent (CD26-) MSTO-CD26 (CD26^+^) JMN CD26-sh^b^ (CD26-) JMN ctrl-sh^b^ (CD26^+^) A549 (CD26-)	**Immunostaining** [Tissue] Liver Kidney Prostate Mesothelioma

^a^ sCD26: soluble human CD26 (the extracellular domain of CD26)

^b^ sh: short hairpin RNA

The host mice, preparation of immunogen, and the immunization method used for our current study were exactly the same as in our previous study [29]. In our previous study, the hybridoma supernatants were screened for selective reactivity with human CD26. As a result of the screening, 31 clones that secreted anti-human CD26 mAbs were evaluated by both flow cytometry and ELISA. After testing the hybridoma supernatants from the 31 clones for immunostaining of CD26 in FFPE tissue sections, we finally obtained 19–32 mAb [29]. On the other hand, in the present study, hybridoma cells were first screened for murine IgG production by ELISA, since it is possible that mAbs suitable for immunostaining of denatured human tumor CD26 in FFPE cell block and tissue specimens would not react well to native (undenatured) CD26 or urea-treated CD26 protein, as analyzed by flow cytometry or ELISA.

### Immunostaining with novel anti-CD26 mAbs

The positive supernatants containing high levels of murine IgG were next screened for immunostaining of CD26 in FFPE cell block of various human CD26-positive or negative tumor cell lines as described above. After testing the hybridoma supernatants from a total of 429 clones for immunostaining of FFPE cell block, we finally obtained 5 clones capable of staining CD26 in CD26-positive tumor cell lines without any non-specific staining in CD26-negative tumor cell lines. Among them, two representative clones (clone U16-3 or U38-8) clearly distinguished CD26-positive cells from CD26-negative cells by immunostaining. As shown in Fig 1A, MSTO-CD26 and JMN ctrl-shRNA cells stained with the purified U16-3 mAb or U38-8 mAb exhibited reliable staining intensity comparable to the control pAb, while MSTO parent, JMN CD26-shRNA and A549 cells stained with these mAbs showed extremely low background signals as compared with the control pAb (panels iii, iv, v). In addition, to determine the optimal Ab concentration for immunostaining, we evaluated the Abs in concentrations ranging from 0.1 μg/ml to 10 μg/ml of U16-3 mAb, U38-8 mAb or control pAb. As a result of this evaluation, staining of FFPE cell block with 0.2–0.5 μg/ml of U16-3 mAb or 0.5–1 μg/ml of U38-8 mAb resulted in similar staining intensity as compared with those stained with 1–2 μg/ml of control pAb (Fig 1A-iii, 1A-iv and 1A-v), strongly suggesting that the newly developed mAbs have a higher binding affinity for denatured human CD26 in FFPE sections than that of control pAb. To further confirm the binding specificity of these novel mAbs to human CD26, the FFPE cell block of MSTO-CD26 and JMN ctrl-shRNA cells was treated with U16-3 mAb or U38-8 mAb preincubated with excessive amounts of native (undenatured) sCD26 or vehicle. As shown in Fig 1B, the binding of these mAbs was completely inhibited by sCD26 (panels i, ii).

**Fig 2:**
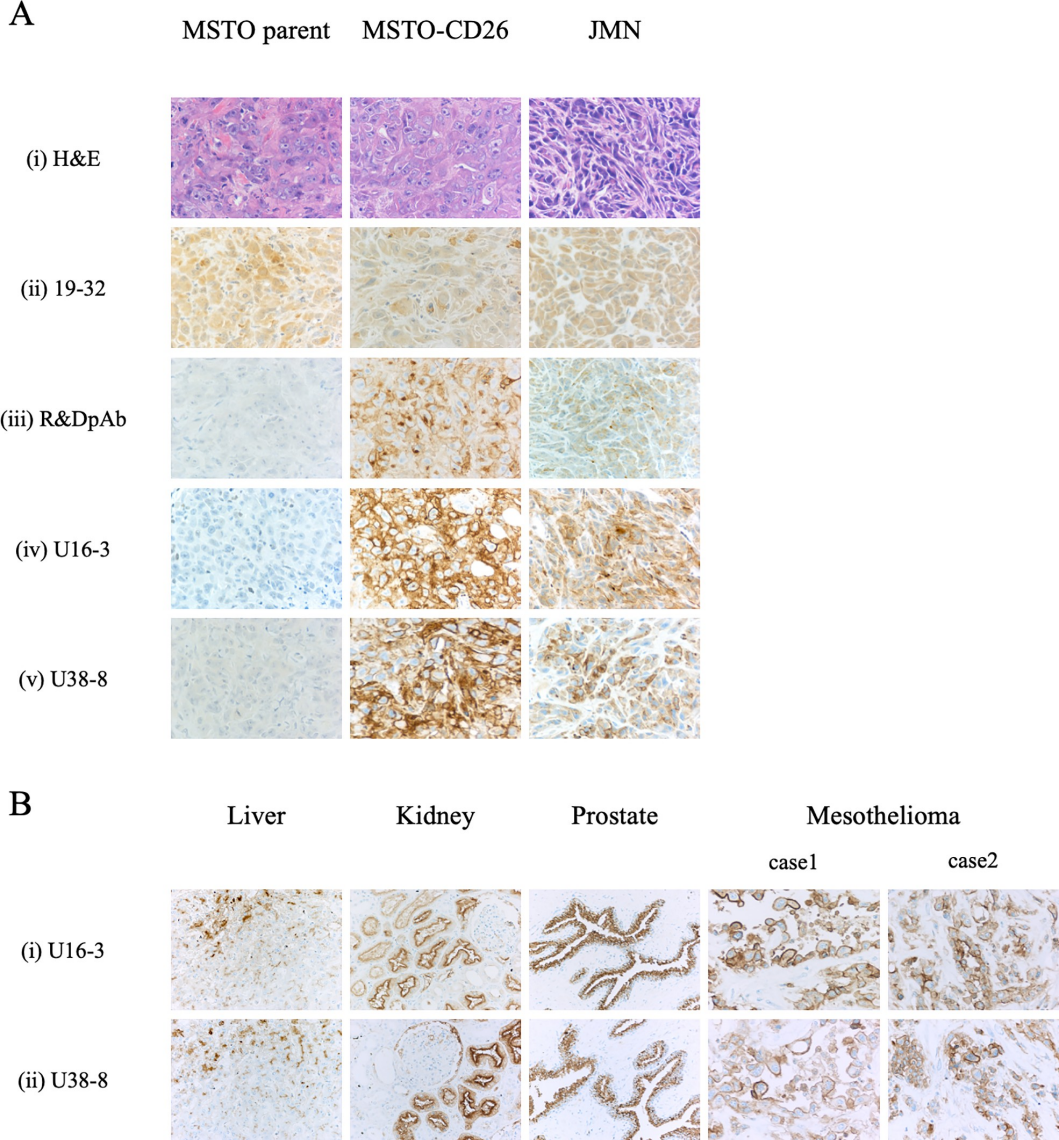
Representative results of immunostaining of FFPE tissue specimens with novel anti-CD26 mAbs. **A.** MSTO parent, MSTO-CD26 or JMN cells were implanted subcutaneously (s.c.) in the flank of SCID mice. The *in vivo* tumor samples were stained with hematoxylin and eosin (H&E) (i), or purified mouse anti-human CD26 mAb (19–32 (ii), U16-3 (iv), U38-8 (v)), or purified goat anti-human CD26 pAb (R&D Systems (iii)). Original magnification, 20x. **B.** The tissue specimens of liver, kidney, prostate or two cases of malignant mesothelioma were stained with purified novel mouse anti-human CD26 mAbs (U16-3 (i) or U38-8 (ii)). Original magnification, 4x. All specimens were counterstained with hematoxylin.

Moreover, we examined immunohistochemical staining of *in vivo* tumor samples with U16-3 mAb or U38-8 mAb. For this purpose, MSTO parent, MSTO-CD26 or JMN cells were implanted s.c. in the flank of SCID mice, and the tumors in the flank were excised from those mice. Histology of mesothelioma formed by MSTO parent, MSTO-CD26 or JMN cells was shown in H&E staining of each tumor sample (Fig 2A-i). Staining of tumors derived from MSTO-CD26 and JMN cells with U16-3 mAb or U38-8 mAb showed more bright staining intensity than control pAb, while no apparent staining was observed in MSTO parent-derived tumors stained with these two mAbs (Fig 2A-iii, 2A-iv and 2A-v). Meanwhile, not only tumors derived from MSTO-CD26 and JMN cells but also tumors derived from MSTO parent cells were all stained with 19–32 mAb (Fig 2A-ii), which was similar with the results of cell block shown in Fig 1A-ii.

Finally, we examined immunohistochemical staining of CD26 in FFPE tissue specimens of normal liver, kidney, prostate, and MPM with U16-3 mAb or U38-8 mAb. The staining pattern of CD26 in these tissues was detailed previously [29]. As shown in Fig 2B, the surface membrane of bile canaliculi, the brush border of renal proximal tubular epithelial cells and prostate epithelial cells were specifically stained with extremely low background (panels i, ii). The specific staining of CD26 in epithelioid type of MPM was also observed with the use of U16-3 mAb or U38-8 mAb. Of note, although both cell surface and cytoplasm of MPM cells were stained with U16-3 mAb or U38-8 mAb, cell surface membrane was stained more intensely than cytoplasm. This staining pattern of CD26 in MPM cells was similar with the staining results of control pAb [29].

Taken together, these data indicate that U16-3 mAb and U38-8 mAb were capable of detecting denatured human CD26 in FFPE non-tumor and tumor tissue sections and tumor cell blocks with reliable staining pattern comparable to the control pAb. Importantly, with respect to signal-to-noise ratio, the newly developed mAbs were superior to the commercial anti-CD26 pAb in immunostaining of FFPE samples.

### Characteristics of novel anti-CD26 mAbs

**Fig 3:**
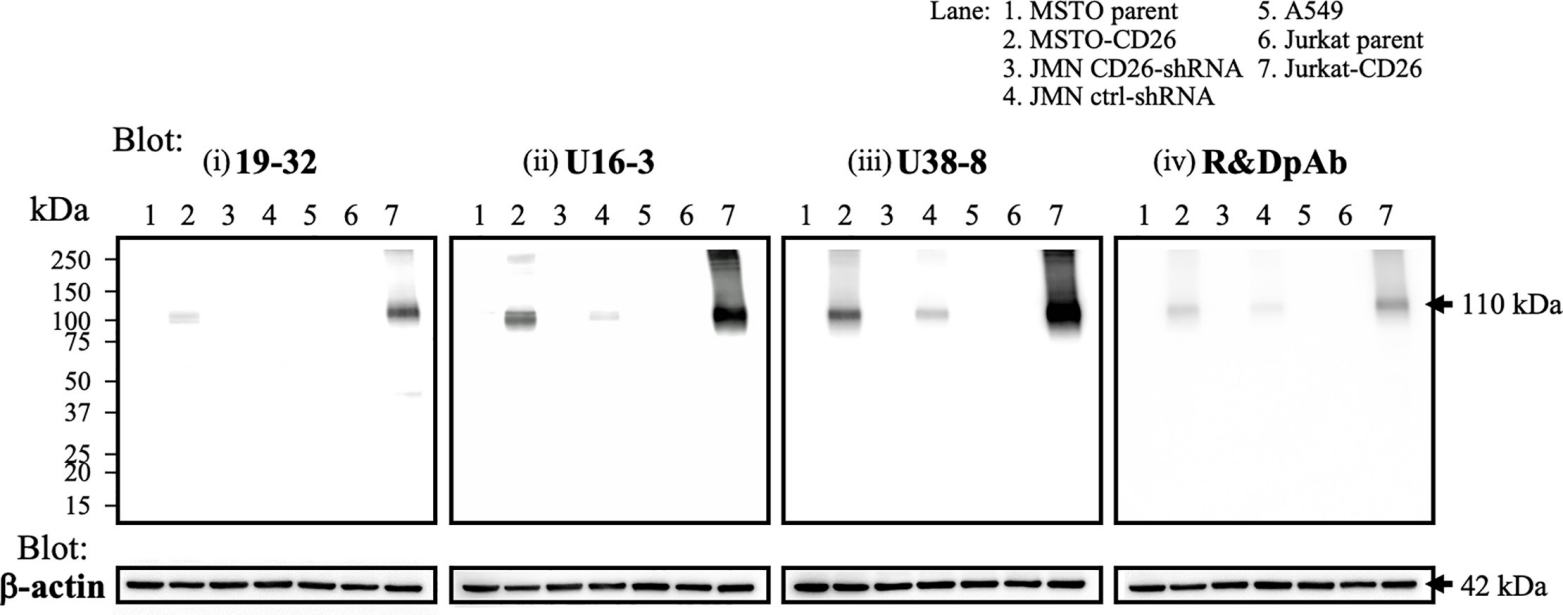
Western blotting analysis with novel anti-CD26 mAbs. Whole cell lysates of MSTO parent, MSTO-CD26, JMN CD26-shRNA, JMN ctrl-shRNA, A549, Jurkat parent or Jurkat-CD26 cells were separated by SDS-PAGE (each, 20 μg), and CD26 was detected by immunoblotting with purified mouse anti-human CD26 mAb (19–32 (i), U16-3 (ii), U38-8 (iii)), or purified goat anti-human CD26 pAb (R&D Systems (iv)). The same blots were stripped and reprobed with antibodies specific for β-actin as a loading control. Data shown are representative of three independent experiments, and similar results were obtained in each experiment.

To further analyze the binding specificity and affinity of novel anti-CD26 mAbs for denatured or undenatured human CD26, we next conducted Western blot analysis utilizing the same human CD26-positive or negative tumor cell lines described above. We used the same enhanced chemiluminescence reagent, and the images were taken at the same exposure time to compare the detection sensitivity of anti-CD26 Abs. As shown in Fig 3, when each sample was immunoblotted with U38-8 mAb, clear bands were detected in the whole cell lysate of MSTO-CD26, JMN ctrl-shRNA and Jurkat-CD26 in molecular mass regions around 110 kDa, while these bands were not observed in the lysate of MSTO parent, JMN CD26-shRNA, A549 or Jurkat parent (panel iii). Importantly, no other non-specific bands were ever observed in all of the samples. The band intensity of Jurkat-CD26 was the most prominent, whereas the band intensity of JMN ctrl-shRNA was considerably weaker compared with those of Jurkat-CD26 and MSTO-CD26. These results correlated with the mRNA and cell surface protein expression levels of human CD26 in these cell lines, as shown in S1 Fig. In addition, U16-3 mAb or the control pAb detected CD26 expression in Jurkat-CD26 and MSTO-CD26, while the CD26 band was only slightly detected in whole cell lysate of JMN ctrl-shRNA (Fig 3-ii and 3-iv). Meanwhile, when each sample was immunoblotted with 19–32 mAb, the clear band was only observed in Jurkat-CD26 (Fig 3-i). When images were obtained at longer exposure times, several non-specific bands in molecular mass regions other than 110 kDa were observed in each sample when immunoblotted with 19–32 mAb. As was the case with immunostaining assay, we evaluated the Abs in concentrations ranging from 0.1 μg/ml to 10 μg/ml of anti-CD26 Abs to determine the optimal Ab concentration for Western blotting. This evaluation demonstrated that immunoblotting with 0.2 μg/ml of U16-3 mAb or 0.5 μg/ml of U38-8 mAb resulted in greater band intensity than that obtained following immunoblotting with 1 μg/ml of control pAb or 5 μg/ml of 19–32 mAb (Fig 3). These results indicate that the newly developed mAbs exhibited significantly greater binding specificity and affinity for denatured human CD26 protein in boiled Western blot samples as compared with those of control pAb or 19–32 mAb.

**Fig 4:**
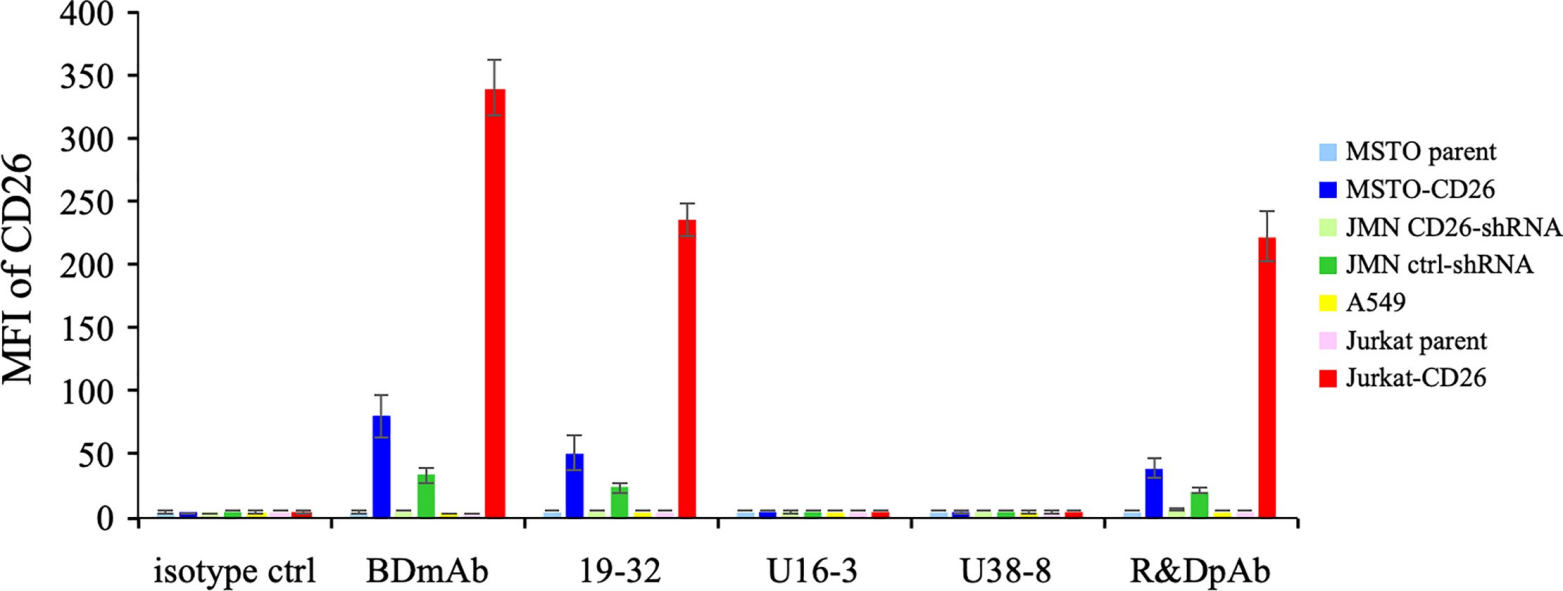
Flow cytometry analysis with novel anti-CD26 mAbs. MSTO parent, MSTO-CD26, JMN CD26-shRNA, JMN ctrl-shRNA, A549, Jurkat parent or Jurkat-CD26 cells were incubated with unlabeled isotype control or purified mouse anti-human CD26 mAb (19–32, U16-3 or U38-8) or purified goat anti-human CD26 pAb (R&D Systems), and subsequently stained with PE-labeled goat anti-mouse Ig pAb or PE-labeled donkey anti-goat IgG Ab, and analyzed by flow cytometry. PE-labeled commercial mouse anti-human CD26 mAb (BD Biosciences, clone M-A261) was utilized as a positive control. Representative data of three independent experiments are shown as mean ± S.D. of the mean fluorescence intensity (MFI) from triplicate samples, and similar results were obtained in each experiment.

We next conducted flow cytometry analysis to analyze the binding affinity of novel anti-CD26 mAbs for native (undenatured) human CD26 protein. Human CD26-positive or negative tumor cell lines were incubated with a sufficient amount (10 μg/ml) of unlabeled mouse anti-human CD26 mAb (19–32, U16-3 or U38-8) or control pAb or isotype controls, and subsequently stained with PE-labeled anti-mouse Ig pAb or PE-labeled anti-goat IgG Ab. PE-labeled commercial anti-CD26 mAb purchased from BD Biosciences or isotype control was utilized as a positive or negative control. As shown in Fig 4 (representative histograms are shown in S2 Fig), anti-CD26 mAb purchased from BD Biosciences, 19–32 mAb or control pAb could stain MSTO-CD26, JMN ctrl-shRNA or Jurkat-CD26 while MSTO parent, JMN CD26-shRNA, A549 or Jurkat parent showed no staining with these anti-CD26 Abs. In contrast, both U16-3 mAb and U38-8 mAb did not stain any of the tumor cell lines, similar to the results obtained with isotype controls (Fig 4). We also evaluated staining with directly fluorochrome-labeled U16-3 mAb or U38-8 mAb, and again these two mAbs did not stain any of the tumor cell lines. These data demonstrate that these novel anti-CD26 mAbs cannot bind to native (undenatured) human CD26 protein on the cell surface.

**Fig 5:**
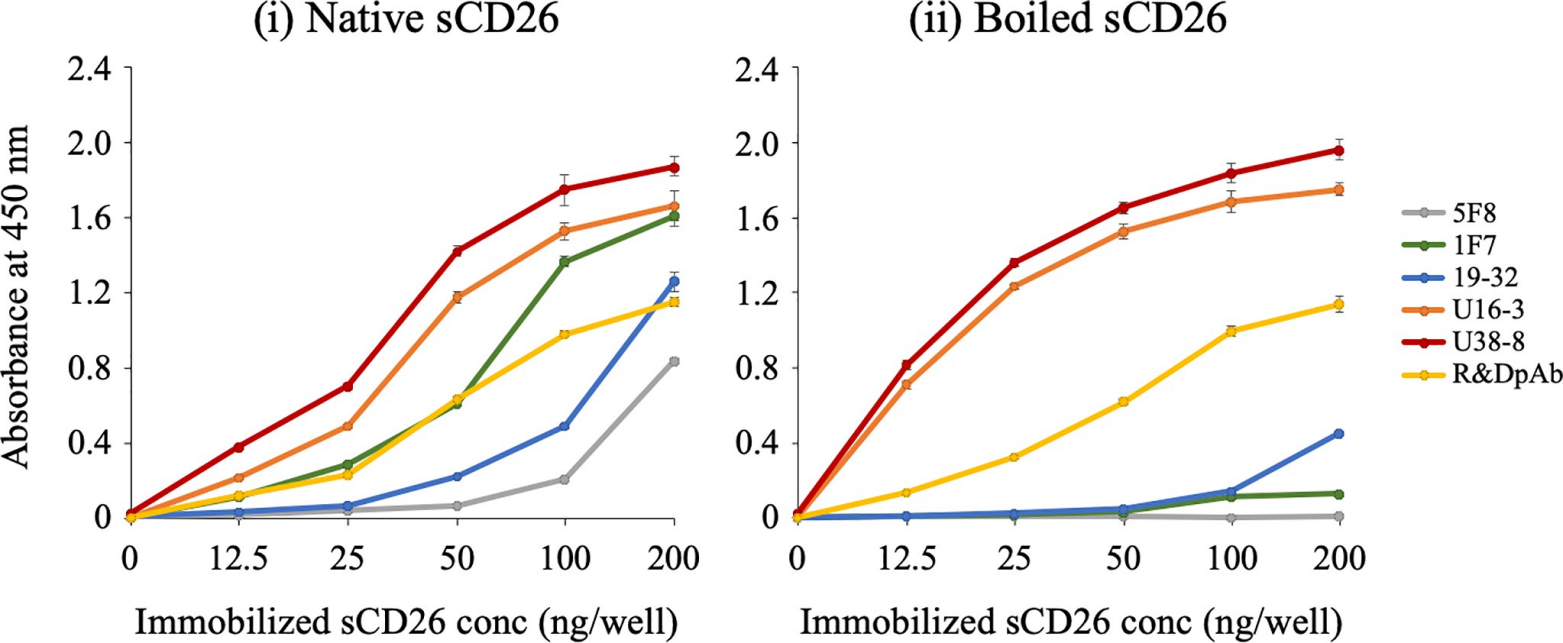
ELISA analysis with novel anti-CD26 mAbs. Immobilized native (undenatured) soluble human CD26 protein (sCD26) (i) or boiled (denatured) sCD26 (ii) was incubated with purified mouse anti-human CD26 mAb (5F8, 1F7, 19–32, U16-3 or U38-8) or purified goat anti-human CD26 pAb (R&D Systems). The absorbance at 450 nm/570 nm was measured. Representative data of three independent experiments are shown as mean ± S.D. of triplicate samples, and similar results were obtained in each experiment.

To further confirm the binding affinity of the novel anti-CD26 mAbs for denatured or undenatured human CD26, we next conducted ELISA assay. To prepare denatured sCD26, purified sCD26 protein was boiled for 10 min at 95°C. The 96-well immunoplates were coated with native (undenatured) sCD26 or denatured sCD26, and the reactivity of anti-CD26 Abs to these proteins was analyzed. Mouse anti-human CD26 mAbs 5F8 and 1F7, which were previously developed by our group as described in *Materials and methods*, were used as controls. Both 5F8 mAb and 1F7 mAb exhibited strong binding affinity to native (undenatured) human CD26 as confirmed by flow cytometry and ELISA, whereas these mAbs did not recognize denatured human CD26 in the FFPE tissue specimens (representative results are shown in S3 Fig). As shown in Fig 5, all the anti-CD26 Abs tested reacted well to the native sCD26, and the absolute value of absorbance was increased in an antigen dose-dependent manner (panel i). On the other hand, when sCD26 was denatured, the absorbance of 5F8 mAb, 1F7 mAb and 19–32 mAb was markedly reduced, while the absorbance of control pAb was maintained (Fig 5-ii). However, intriguingly, the absolute value of absorbance to the denatured sCD26 incubated with U16-3 mAb or U38-8 mAb appeared to be higher than the absorbance to the native sCD26 incubated with U16-3 mAb or U38-8 mAb, and the observed difference was particularly prominent at the low antigen doses (Fig 5). Moreover, the absolute value of absorbance to the denatured sCD26 incubated with U16-3 mAb or U38-8 mAb was much higher than that of control pAb. We next evaluated the potential suitability of U16-3 mAb or U38-8 mAb for sandwich ELISA. For this purpose, we selected 5 mouse anti-human CD26 mAbs (19–32, 1F7, 5F8, 16D4B or 9C11) and a humanized anti-CD26 mAb (YS110) which were all previously developed in our laboratory. We have previously shown that these mAbs recognized distinct CD26 regions [26, 29]. All of these mAbs were labeled with biotin or HRP, and we tested all possible combinations of U16-3 mAb and other mAb, or U38-8 mAb and other mAb. However, the absolute value of absorbance was nearly at blank level for all of the combinations, suggesting that both U16-3 mAb and U38-8 mAb are not suitable for sandwich ELISA, and that these two mAbs hardly recognize native sCD26 in the liquid phase.

Taken together, our results obtained from immunostaining, Western blotting, flow cytometry and ELISA assays demonstrate that the newly developed mAbs exhibit significant binding specificity and affinity for denatured human CD26 protein in FFPE cell block and tissue specimens or boiled Western blot samples and recombinant sCD26 protein, as compared with those of control pAb or 19–32 mAb.

### Advances in future CD26-related research

**Fig 6:**
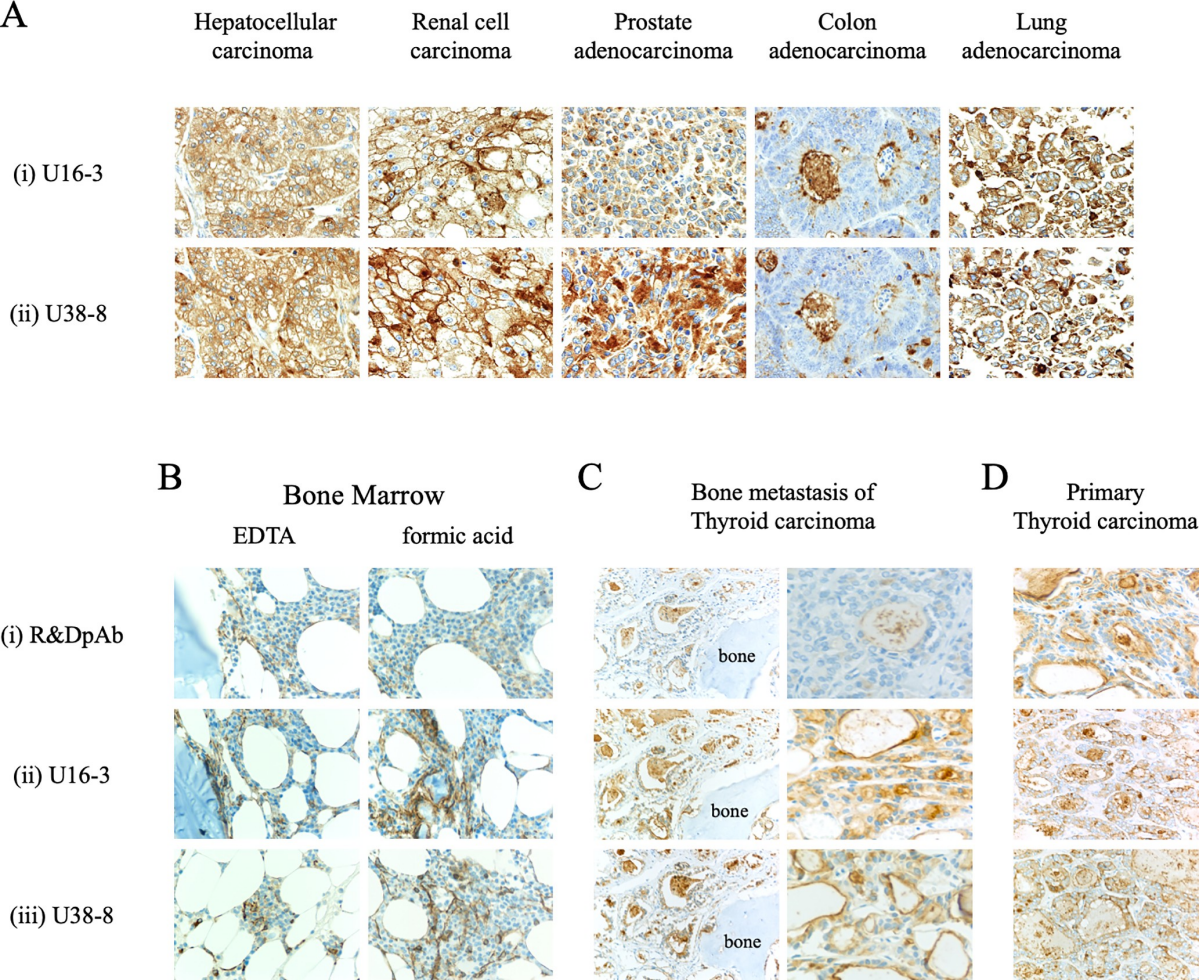
Novel anti-CD26 mAbs exhibit a reliable staining pattern and intensity for various tumors and decalcified specimens. **A.** The tissue specimens of hepatocellular carcinoma, renal cell carcinoma, prostate adenocarcinoma, colon adenocarcinoma or lung adenocarcinoma were stained with purified novel mouse anti-human CD26 mAbs (U16-3 (i) or U38-8 (ii)). Original magnification, 40x. **B, C, D.** The EDTA-decalcified (left panels) or formic acid-decalcified (right panels) tissue specimens of normal human bone and bone marrow (B), formic acid-decalcified tissue specimens of metastatic thyroid carcinoma in the bone (C), or the tissue specimens of primary thyroid carcinoma without decalcification (D) were stained with purified goat anti-human CD26 pAb (R&D Systems (i)) or purified novel mouse anti-human CD26 mAbs (U16-3 (ii) or U38-8 (iii)). Original magnification, 10x (B, left panels of C, D) or 40x (right panels of C). All specimens were counterstained with hematoxylin.

Since our data reveal that the newly developed anti-CD26 mAbs can specifically stain denatured human CD26 in FFPE tumor cells with reliable clarity and intensity, we then conducted immunohistochemical staining of FFPE CD26-expressing tumor tissues of hepatocellular carcinoma, renal cell carcinoma, prostate adenocarcinoma, colon adenocarcinoma, and lung adenocarcinoma with U16-3 mAb or U38-8 mAb. As shown in Fig 6A, each tumor tissue stained with U16-3 mAb or U38-8 mAb (panels i and ii) exhibited stronger and broader staining patterns as compared with staining with control pAb [29]. Results from the immunostaining studies indicate that CD26 can be detected both on the cell surface as well as in the cytoplasm of these carcinoma tissues.

Staining of decalcified specimens with reliable clarity and intensity is well-known to be challenging since proteins are further denatured and/or degenerated in the process of decalcification. Since our results demonstrate that the novel anti-CD26 mAbs have a higher binding affinity for denatured human CD26 protein than that of commercial anti-CD26 pAb (Figs 1, 2, 3 and 5), we proceeded to conduct immunohistochemical staining of decalcified specimens with U16-3 mAb or U38-8 mAb. As shown in Fig 6B, both EDTA-decalcified and formic acid-decalcified normal human bone and bone marrow tissue specimens stained with U16-3 mAb or U38-8 mAb exhibited a bright staining pattern of CD26, with even the distinct vasculature of marrow being specifically stained with reliable intensity, while control pAb could not stain decalcified bone tissue with reliable clarity and intensity (panels i, ii, iii). Moreover, we stained decalcified specimens of metastatic thyroid carcinoma in the bone. CD26 expression was clearly visible on the cell surface and in the cytoplasm of carcinoma cells when stained with U16-3 mAb or U38-8 mAb following decalcification (Fig 6C-ii and 6C-iii). On the other hand, staining of decalcified specimens with control pAb was only faintly observed in colloid space whereas bright staining was observed in the specimens of primary thyroid carcinoma without decalcification (Fig 6C-i and 6D-i). Taken together, these data strongly suggest that the newly developed anti-CD26 mAbs are potentially useful as companion diagnostic agents to analyze tumor CD26 expression in the clinical setting, while advancing future CD26-related research.

## Discussion

Although it is difficult to develop anti-human CD26 mAbs that can clearly detect denatured CD26 in FFPE tissues, the anti-CD26 pAb purchased from R&D Systems is able to stain CD26 with reliable clarity and intensity. However, since treatment with targeted therapeutic agents is dependent on detection of the appropriate target antigens on clinical samples, uniformity of the diagnostic reagents is critical, suggesting that mAbs are desirable for diagnostic uses in the clinical setting. In the present study, we have improved the screening methods and succeeded in developing novel anti-human CD26 mAbs that can potentially be used as diagnostic reagents clinically.

CD26 is highly expressed on the surface of MPM cells, especially tumors of the epithelioid and biphasic types, but not on benign mesothelial tissues [21, 28]. We have previously evaluated the prognostic significance of CD26 membrane expression on MPM cells and other clinicopathological factors in those patients, and concluded that the CD26 molecule is a reliable biomarker for predicting potential therapeutic outcome of MPM patients following chemotherapy [28]. Our previous study showed that CD26 is associated with high proliferative activity and invasiveness of MPM cells [22, 23, 28]. Although the exact role of CD26/DPPIV in other cancers remains to be elucidated, CD26 serves as a prognostic marker in multiple tumors. Significantly higher CD26 expression has been shown to be correlated with poorly differentiated CRC, late tumor node metastasis stage, and development of metastasis [10]. In addition, a high CD26 expression level is a predictor of poor outcome after resection of CRC. The disease-free survival of patients with CD26-positive GIST of the stomach is worse than that of patients with CD26-negative GIST [11]. CD26, secretogranin V (SCG5) and carbonic anhydrase XII (CA12) are a three-gene signature that can distinguish malignant thyroid cancers, and is useful for preoperative diagnosis of thyroid cancer [12]. The mRNA level of CD26 is significantly upregulated in advanced-stage urothelial carcinoma and the upregulation of CD26 is most significantly associated with clinical aggressiveness [13]. CD26 expression level in prostate cancer tissues is higher than that of normal prostatic tissues, and is enhanced with prostate cancer stage advancement [14]. These observations strongly suggest that immunohistochemical staining of CD26 in FFPE tumor tissues is important for diagnosis and prognosis of multiple tumors. Also, more detailed analysis regarding the localization and quantification of CD26 expression may provide additional benefit in the clinical setting.

Specific binding with its target molecule is a well-known characteristic of antibody. However, in Western blot assay, several non-specific bands may often be observed in each sample when immunoblotted with commercial Abs. Similarly, a FFPE negative control sample is often non-specifically stained with the Abs. Under denaturing conditions, the conformation of proteins is changed, and conformational amino acid sequences that do not exist in the physiological condition are generated. If the generated amino acid sequences are similar to the epitope of the Abs, these Abs react with several proteins in the denaturing conditions. Different denaturing conditions such as FFPE treatment, urea treatment and boiling may result in different conformational amino acid sequences. Our previous 19–32 mAb was first screened for selective reactivity with native (undenatured) human CD26 by flow cytometry and ELISA, and we confirmed that 19–32 mAb did not stain Jurkat parent cells by flow cytometry and did not react with blocking protein by ELISA (Fig 4, S2 Fig, Fig 5-i). In addition, we have conducted immunohistochemical staining of nearly one hundred FFPE MPM tissue specimens with 19–32 mAb, and found that not all of the MPM samples were stained with 19–32 mAb. However, on the other hand, FFPE cell block specimens of CD26-negative tumor cell lines were all unexpectedly stained with 19–32 mAb (Fig 1A-ii), suggesting that 19–32 mAb binds with human CD26 as well as denatured antigen(s) commonly expressed in tumor cell lines. Selection of screening methods is crucially important for the development of novel mAb designed to serve specific purpose. In the present study, hybridomas producing high levels of murine IgG were all screened for immunostaining of FFPE cell blocks. From a total of 429 clones, we obtained only 5 clones capable of distinguishing CD26-positive cells from CD26-negative cells by immunostaining. These screening methods utilizing a set of various human CD26-positive or negative tumor cell lines are useful for selecting mAbs that specifically recognize denatured CD26 with a high signal-to-noise ratio.

Meanwhile, results from flow cytometry showed that both U16-3 mAb and U38-8 mAb did not bind to native (undenatured) human CD26 on the cell surface (Fig 4, S2 Fig). Moreover, although both U16-3 mAb and U38-8 mAb exhibited strong binding affinity to the immobilized native sCD26 protein (Fig 5-i), these two mAbs barely recognized native sCD26 protein in the liquid phase as confirmed by sandwich ELISA. In contrast, the antibody-absorption test showed that staining of MSTO-CD26 and JMN ctrl-shRNA cells with U16-3 mAb or U38-8 mAb was completely inhibited by native sCD26 protein (Fig 1B). In that experiment, 6.67 pmol of U16-3 mAb or U38-8 mAb was preincubated with 909 pmol of native sCD26 protein. More than one hundred times larger amount of antigen was needed for the antibody-absorption test, suggesting that these two mAbs did not react well with native sCD26 protein. Although the precise difference in the conformation of CD26 protein on the cell surface and that of sCD26 protein in the liquid phase is not elucidated, our data strongly suggest that these novel anti-CD26 mAbs do not have strong binding affinity to native human CD26. We have previously shown that the epitopes of human CD26 defined by anti-CD26 mAbs were roughly categorized by 5 separate groups utilizing human CD26 deletion mutant- or human-rat CD26 swap mutant-transfected cells [26]. Cross-blocking studies such as flow cytometry or sandwich ELISA using several anti-CD26 mAbs, the binding regions of which were already characterized, enabled us to speculate on the epitope defined by 19–32 mAb [29]. However, both U16-3 mAb and U38-8 mAb cannot be used for flow cytometry and sandwich ELISA. Moreover, it is quite difficult to prepare denatured CD26 protein that can exactly replicate the conformation of CD26 in FFPE section or in decalcified tissue section. These limitations have limited our ability to analyze the precise epitopes defined by the novel anti-CD26 mAbs. However, accumulating evidences regarding the conformational change of denatured proteins are found in the protein database, and we have already obtained data regarding the amino acid sequence of the variable region in both heavy chain and light chain of U16-3 mAb and U38-8 mAb, utilizing the 5’-RACE PCR method. We are currently examining *in silico* predictions of the three-dimensional structure of an antigen-antibody complex utilizing such program as Rosetta [32].

The mAbs capable of staining target molecules with reliable clarity and intensity in decalcified specimens are valuable. We have recently reported that CD26 is expressed on normal human osteoclasts, with its expression being enhanced following activation [33]. CD26 expression is increased in the process of osteoclast differentiation, and may be involved in p38 MAPK signaling. Interestingly, the humanized anti-CD26 mAb (YS110) inhibits early osteoclast precursor differentiation into osteoclasts [33]. Since our present data indicate that both U16-3 mAb and U38-8 mAb can clearly stain CD26 in the bone or on tumor cells in calcified tissues (Fig 6B and 6C), these mAbs are expected to contribute to future CD26-related research involving normal tissues and tumor/inflammatory lesions accompanied by calcification. Furthermore, these mAbs are potentially useful for the analysis of CD26 expression in cancer patients with bony metastasis, and may help decide the appropriateness of YS110 therapy for future cancer patients.

## Supporting information

S1 Fig mRNA and cell surface protein expression of CD26 in the human tumor cell lines used in this study. **A.** Total RNA was extracted from the indicated cell lines by the use of Rneasy Mini Kit according to the manufacturer’s instructions (QIAGEN, Valencia, CA), and cDNA was produced by using PrimeScript II 1st strand cDNA Synthesis Kit (TaKaRa Bio, Shiga, Japan) with oligo dT primer. Quantification of mRNA was performed using the 7500 Real-Time PCR System and SYBR Select Master Mix (Applied Biosystems, Foster City, CA). The obtained data were analyzed with 7500 System SDS Software (Applied Biosystems), being normalized to hypoxanthine phosphoribosyltransferase 1 (HPRT1) expression. The PCR was performed using the following primers: CD26 forward primer, 5’-GTACACAGAACGTTACATGGGTCTC-3’; reverse primer, 5’-TCAGCTCTGCTCATGACTGTTG-3’; HPRT1 forward primer, 5’-CAGTC AACAGGGGACATAAAAG-3’; reverse primer, 5’-CCTGACCAAGGAAAGCAAAG-3’. Data are shown as mean ± S.D. of triplicate samples.
**B.** The indicated cells were incubated with PE-labeled isotype control (BD Biosciences, clone MOPC-21 (i)) or PE-labeled commercial mouse anti-human CD26 mAb (BD Biosciences, clone M-A261 (ii)), and cell surface expression of CD26 was analyzed by flow cytometry. Two-dimensional dot plot (horizontal axis: CD26, longitudinal axis: non-staining) gated for viable cells is shown. A representative plot of three independent experiments is shown, and similar results were obtained with each experiment.
Among cell lines used in this study, mRNA and cell surface protein expression of CD26 in Jurkat-CD26 is the most prominent, and the expression levels of MSTO-CD26 are higher than those of JMN ctrl-shRNA cells, whereas CD26 is hardly expressed even at mRNA level in MSTO parent, JMN CD26-shRNA, A549 and Jurkat parent.
(PDF)

S2 Fig Representative results of flow cytometry with novel anti-CD26 mAbs. MSTO parent, MSTO-CD26, JMN CD26-shRNA, JMN ctrl-shRNA, A549, Jurkat parent or Jurkat-CD26 cells were incubated with unlabeled isotype control or purified mouse anti-human CD26 mAb (19–32 (ii), U16-3 (iii) or U38-8 (iv)) or purified goat anti-human CD26 pAb (R&D Systems (v)), and subsequently stained with PE-labeled goat anti-mouse Ig pAb or PE-labeled donkey anti-goat IgG Ab, and analyzed by flow cytometry. PE-labeled commercial mouse anti-human CD26 mAb (BD Biosciences, clone M-A261 (i)) was utilized as a positive control. Data are shown as histogram of CD26 intensity (red lines), and the gray area in each histogram shows data of the isotype control. A representative histogram of three independent experiments is shown, and similar results were obtained in each experiment.
PE-labeled anti-CD26 mAb purchased from BD Biosciences, 19–32 mAb or anti-CD26 pAb purchased from R&D Systems could stain MSTO-CD26, JMN ctrl-shRNA or Jurkat-CD26 while MSTO parent, JMN CD26-shRNA, A549 or Jurkat parent did not demonstrate staining with these anti-CD26 Abs. In contrast, both U16-3 mAb and U38-8 mAb did not stain any of the tumor cell lines (similar to results seen with isotype controls).
(PDF)

S3 Fig Immunohistochemical staining with anti-CD26 mAbs incapable of detecting denatured human CD26. The tissue specimens of liver, kidney, prostate or two cases of malignant mesothelioma were stained with purified mouse anti-human CD26 mAbs (1F7 (i) or 5F8 (ii)), which were previously developed in our laboratory. Original magnification, 4x. All specimens were counterstained with hematoxylin.
No apparent staining of CD26 was observed in the formalin-fixed paraffin-embedded tissue specimens stained with 1F7 mAb or 5F8 mAb.
(PDF)

## Abbreviations

AAs: amino acids
Ab: antibody
CRC: colorectal cancer
DPPIV: dipeptidyl peptidase IV
ELISA: enzyme-linked immunosorbent assay
FFPE: formalin-fixed paraffin-embedded
FIH: first-in-human
GIST: gastrointestinal stromal tumor
HRP: horseradish peroxidase
mAb: monoclonal antibody
MPM: malignant pleural mesothelioma
pAb: polyclonal antibody
RCC: renal carcinoma
sCD26: soluble human CD26
shRNA: short hairpin RNA.
