# Supplementary figures and images for: Development of novel monoclonal antibodies with specific binding affinity for denatured human CD26 in formalin-fixed paraffin-embedded and decalcified specimens
Source: PLoS One. 2019 Jun 13;14(6):e0218330. doi: 10.1371/journal.pone.0218330 (PMC6564021; doi:10.1371/journal.pone.0218330)

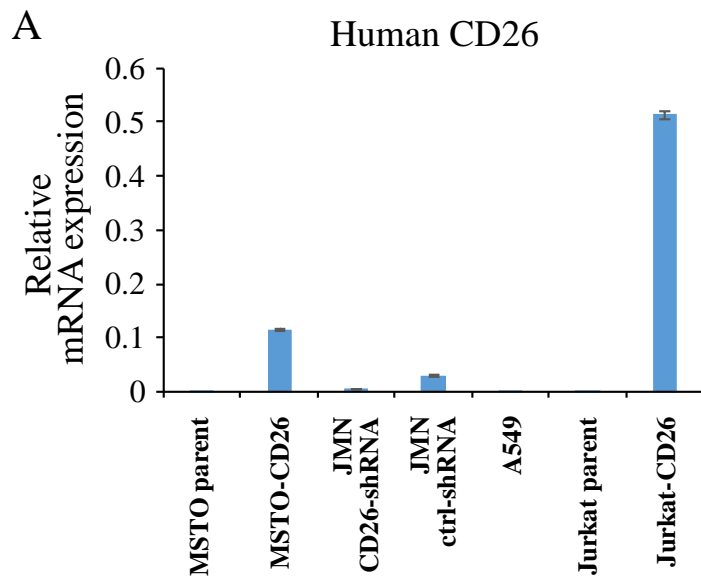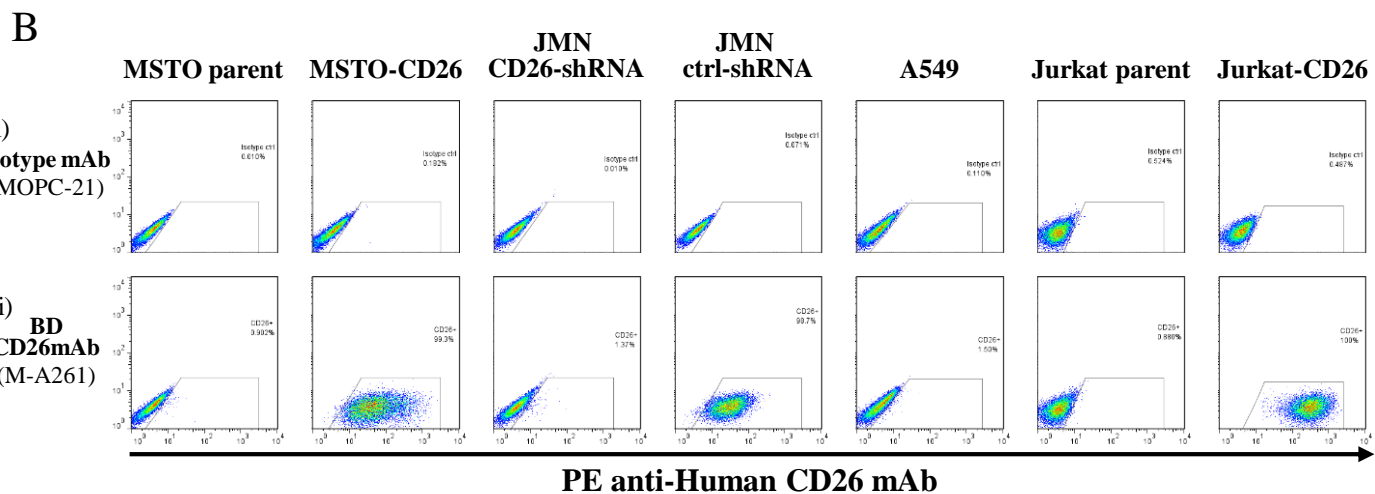

Supplement: S1 Fig — A. Total RNA was extracted from the indicated cell lines by the use of Rneasy Mini Kit according to the manufacturer’s instructions (QIAGEN, Valencia, CA), and cDNA was produced by using PrimeScript II 1st strand cDNA Synthesis Kit (TaKaRa Bio, Shiga, Japan) with oligo dT primer. Quantification of mRNA was performed using the 7500 Real-Time PCR System and SYBR Select Master Mix (Applied Biosystems, Foster City, CA). The obtained data were analyzed with 7500 System SDS Software (Applied Biosystems), being normalized to hypoxanthine phosphoribosyltransferase 1 (HPRT1) expression. The PCR was performed using the following primers: CD26 forward primer, 5’-GTACACAGAACGTTACATGGGTCTC-3’; reverse primer, 5’-TCAGCTCTGCTCATGACTGTTG-3’; HPRT1 forward primer, 5’-CAGTC AACAGGGGACATAAAAG-3’; reverse primer, 5’-CCTGACCAAGGAAAGCAAAG-3’. Data are shown as mean ± S.D. of triplicate samples. B. The indicated cells were incubated with PE-labeled isotype control (BD Biosciences, clone MOPC-21 (i)) or PE-labeled commercial mouse anti-human CD26 mAb (BD Biosciences, clone M-A261 (ii)), and cell surface expression of CD26 was analyzed by flow cytometry. Two-dimensional dot plot (horizontal axis: CD26, longitudinal axis: non-staining) gated for viable cells is shown. A representative plot of three independent experiments is shown, and similar results were obtained with each experiment. Among cell lines used in this study, mRNA and cell surface protein expression of CD26 in Jurkat-CD26 is the most prominent, and the expression levels of MSTO-CD26 are higher than those of JMN ctrl-shRNA cells, whereas CD26 is hardly expressed even at mRNA level in MSTO parent, JMN CD26-shRNA, A549 and Jurkat parent. (PDF) [file pone.0218330.s001.pdf]

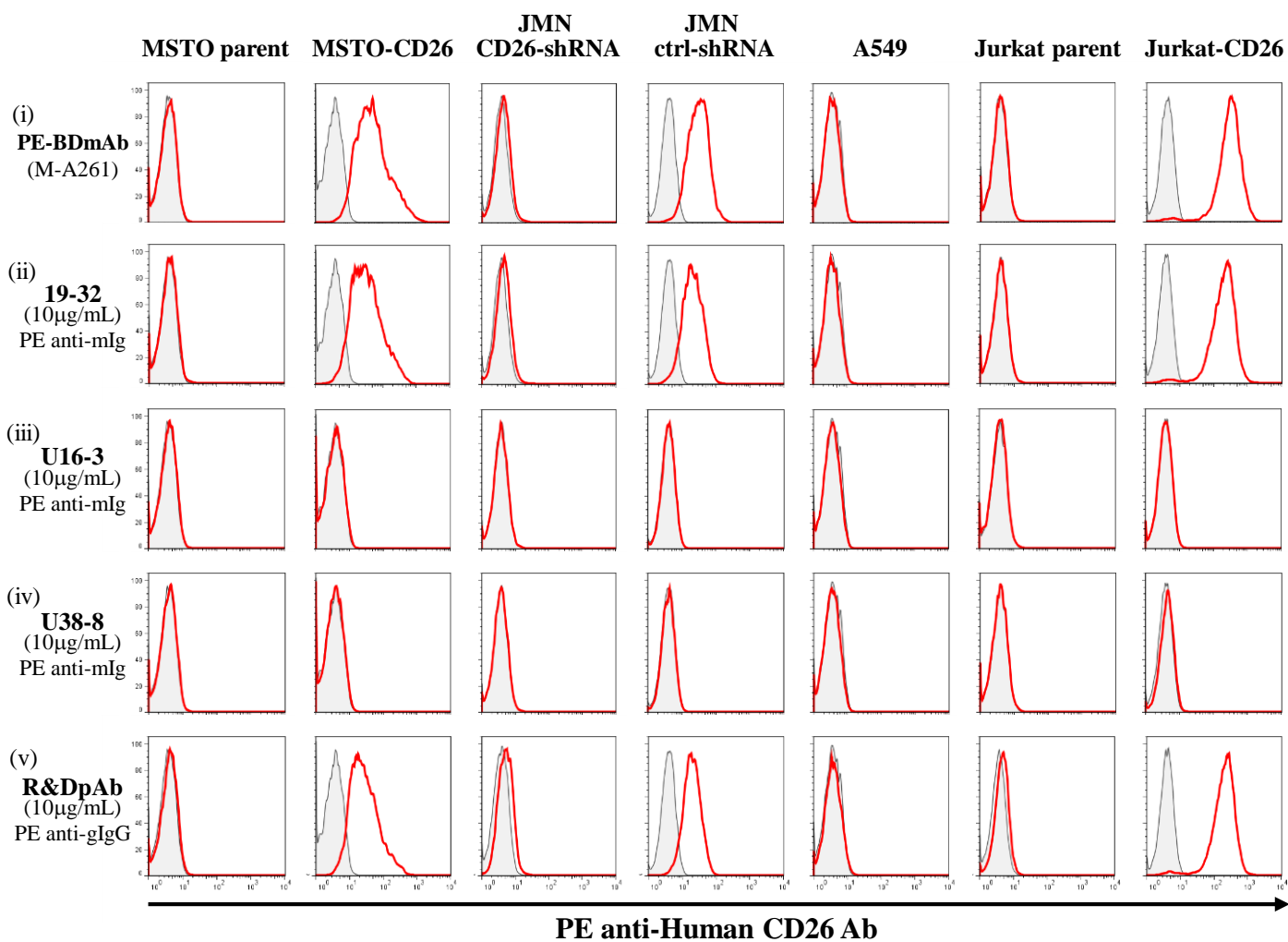

Supplement: S2 Fig — MSTO parent, MSTO-CD26, JMN CD26-shRNA, JMN ctrl-shRNA, A549, Jurkat parent or Jurkat-CD26 cells were incubated with unlabeled isotype control or purified mouse anti-human CD26 mAb (19–32 (ii), U16-3 (iii) or U38-8 (iv)) or purified goat anti-human CD26 pAb (R&D Systems (v)), and subsequently stained with PE-labeled goat anti-mouse Ig pAb or PE-labeled donkey anti-goat IgG Ab, and analyzed by flow cytometry. PE-labeled commercial mouse anti-human CD26 mAb (BD Biosciences, clone M-A261 (i)) was utilized as a positive control. Data are shown as histogram of CD26 intensity (red lines), and the gray area in each histogram shows data of the isotype control. A representative histogram of three independent experiments is shown, and similar results were obtained in each experiment. PE-labeled anti-CD26 mAb purchased from BD Biosciences, 19–32 mAb or anti-CD26 pAb purchased from R&D Systems could stain MSTO-CD26, JMN ctrl-shRNA or Jurkat-CD26 while MSTO parent, JMN CD26-shRNA, A549 or Jurkat parent did not demonstrate staining with these anti-CD26 Abs. In contrast, both U16-3 mAb and U38-8 mAb did not stain any of the tumor cell lines (similar to results seen with isotype controls). (PDF) [file pone.0218330.s002.pdf]

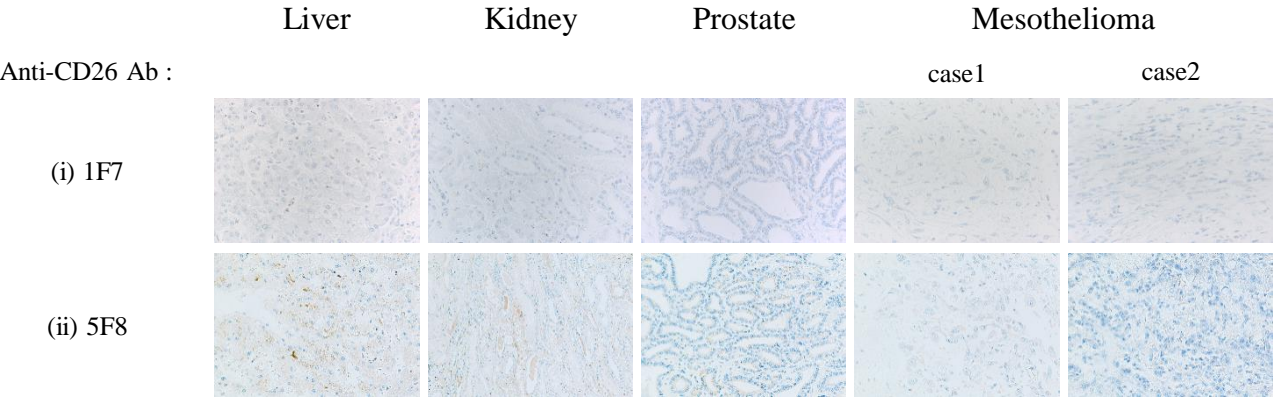

Supplement: S3 Fig — The tissue specimens of liver, kidney, prostate or two cases of malignant mesothelioma were stained with purified mouse anti-human CD26 mAbs (1F7 (i) or 5F8 (ii)), which were previously developed in our laboratory. Original magnification, 4x. All specimens were counterstained with hematoxylin. No apparent staining of CD26 was observed in the formalin-fixed paraffin-embedded tissue specimens stained with 1F7 mAb or 5F8 mAb. (PDF) [file pone.0218330.s003.pdf]
